# The mycotoxin deoxynivalenol (DON) can deteriorate vaccination efficacy against porcine reproductive and respiratory syndrome virus (PRRSV) at subtoxic levels

**DOI:** 10.1186/s40813-022-00254-1

**Published:** 2022-03-20

**Authors:** Antje Rückner, Lisa Plagge, Kristin Heenemann, Maxi Harzer, Bastian Thaa, Janine Winkler, Sven Dänicke, Johannes Kauffold, Thomas W. Vahlenkamp

**Affiliations:** 1 Centre for Infectious Diseases, Institute of Virology, Faculty of Veterinary Medicine, University of Leipzig, An den Tierkliniken 29, 04103 Leipzig, Germany; 2 Institute of Animal Nutrition, Friedrich Loeffler Institute, Federal Research Institute for Animal Health, Brunswick, Germany; 3 Clinic for Ruminants and Swine, Faculty of Veterinary Medicine, University of Leipzig, Leipzig, Germany

**Keywords:** Porcine reproductive and respiratory syndrome virus, Arterivirus, Modified live vaccine, Deoxynivalenol, Mycotoxin, Vaccination, Challenge

## Abstract

**Background:**

Feedgrain contamination with mycotoxins, including deoxynivalenol (DON, “vomitoxin”) is relatively frequently encountered. Pigs are particularly sensitive to the toxicity of DON. To assess the interplay between DON and porcine reproductive and respiratory syndrome virus (PRRSV), we performed an experimental DON exposure–PRRSV vaccination–challenge infection trial. Three-week-old piglets were divided into four groups. Groups I, II and III (10 animals/group) were vaccinated with a PRRSV modified live vaccine and 2 weeks later challenged with a heterologous field strain. While group I was not supplemented with DON, animals in groups II and III received DON for 4 weeks prior to challenge infection at levels that can be encountered in pig feed, employing a low-dose or high-dose regime (group II: 40 µg DON/kg body weight per day; group III: 80 µg DON/kg body weight per day, corresponding to approx. 1 or 2 mg DON/kg feed, respectively). Eight animals (group IV; unvaccinated, not DON exposed) served as control animals for the challenge infection.

**Results:**

We assessed clinical signs, virus load in serum and various organs as well as antibody titres in the animals. All vaccinated animals mounted an efficient PRRSV-specific antibody response within 2 weeks, except for 20% of the animals receiving the higher DON dose. Upon virus challenge, the vaccinated animals in group I were protected from clinical signs. Vaccinated DON-exposed animals in group II and III were protected from clinical signs to a lesser extent. Clinical signs in group III receiving the higher dose of DON were as severe as in the (unvaccinated, not DON exposed) control group IV. The animals of group III also displayed lower antibody titres compared with the animals in group I and II.

**Conclusions:**

The experimental vaccination/challenge study therefore revealed that exposure of pigs to DON for a period of 4 weeks deteriorates the efficacy of vaccination against clinical signs of PRRS.

## Background

Cereals such as wheat, barley and corn are often contaminated with mycotoxins, produced as secondary metabolites by common molds such as *Fusarium* [1]. Of these mycotoxins, the trichothecene deoxynivalenol (DON) is among the most prevalent worldwide. It is formed upon fungal infection of cereals before harvest, particularly under moist weather conditions, and is therefore often present in crop commodities, significantly affecting their quality [2]. Among domestic animals, pigs are considered particularly sensitive to DON toxicity, more so than ruminants or poultry [3]. High concentrations of DON (> 20 mg/kg feed) cause acute symptoms such as intestinal lesions, diarrhoea and vomiting, hence DON’s designation as “vomitoxin” [4]. Chronic exposure to low levels of DON (up to around 2 mg/kg feed) is more common, typically leading to reduced feed consumption (anorexia), decreased nutritional efficiency and hence reduced weight gain. Feed contamination with up to 0.9 mg DON/kg (dry matter) is considered safe for pigs under normal production conditions [5].

It has been described that DON induces some degree of immune modulation. On the molecular level, DON toxicity is caused by inhibition of protein translation *via* binding to the large ribosomal subunit—a mode of action which could inhibit immune actions and/or trigger stress responses [6, 7]. On the cellular level in porcine systems, DON exposure induces changes in cellular signal transduction (principally activation of mitogen-activated protein kinase (MAPK) signalling) as well as alterations in cytokine expression, in particular an increase in pro-inflammatory tumour necrosis factor (TNF) α and interleukin-1β levels [8–10]. Immunoglobulin dysregulation towards higher levels of IgA was reported in DON-exposed mice, but not consistently in pigs [2]. Conflicting results were reported regarding in vivo cytokine profiles [9] or the response to immunisation with model antigens [11, 12] in pigs upon experimental exposure to DON. Generally, no definite conclusions on DON’s immunomodulatory roles can be drawn as the mycotoxin can be suppressive or stimulatory, dependent on duration, frequency and dose of exposure as well as metabolic adaptation [3, 13, 14].

It is also largely unexplored whether and how prolonged DON exposure affects the response to pathogens especially in the pig. In this study, we explored the effects of DON with respect to porcine reproductive and respiratory syndrome (PRRS) virus. PRRSV is an enveloped virus with a positive-stranded RNA genome grouped in the family *Arteriviridae*, order *Nidovirales* [15]. Infection with this virus causes reproductive failure in pregnant sows (abortions, mummified foetuses, stillbirth) as well as respiratory disease and therefore poor growth performance in piglets [16]. PRRS is the swine disease with the highest economic impact in pig-producing countries worldwide, accounting for annual losses of at least $600 million in the USA alone [17]. The virus was identified in the late 1980s in Europe and North America in two distinct genotypes, now divided into the PRRSV-1 (European) and PRRSV-2 (North American) species. Since then, both species have spread worldwide. PRRSV has diversified rapidly by mutation and recombination, including the occurrence of highly pathogenic variants in China ([18], PRRSV-2), North America ([19], PRRSV-2) and Eastern Europe ([20], PRRSV-1).

Due to heterogeneity of PRRSV field strains, a number of PRRSV vaccines—mostly modified live vaccines (MLV)—have been licensed and are in use today [21]. Protective immune responses against PRRSV are difficult to achieve because of viral diversity and also due to the apparent failure of the porcine immune system to mount an immediate and robust adaptive (humoral and cellular) immune response [22, 23].

**Fig. 1:**
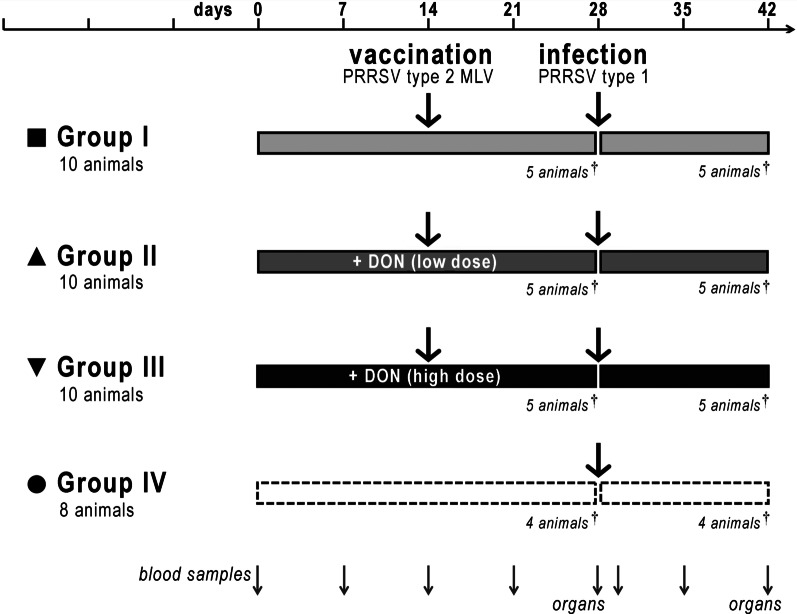
Outline of the animal trial. *Group I* (10 piglets) was vaccinated on day 14 after weaning with the PRRSV-2 based Ingelvac® PRRS MLV (Boehringer-Ingelheim) and infected with the PRRSV-1 field isolate Cobbelsdorf on day 28 (i.e., 14 days post-vaccination). *Group II* (10 piglets) was vaccinated and challenged in the same manner, but additionally given 40 µg DON/kg body weight (“low dose”) on a daily basis on days 1–28. *Group III* (10 piglets) was treated analogously, but supplied with 80 µg DON/kg body weight (“high dose”). Animals in *group IV* (control group, 8 piglets) were mock-vaccinated on day 14, infected on day 28 and did not receive additional DON. Animals were monitored daily and euthanised at the indicated times; blood and organ samples were taken as displayed. For details, see text

We hypothesised that the exposure of pigs to the mycotoxin DON may further hamper the induction of protective immunity by a PRRSV MLV. To address this issue experimentally, we performed an animal trial involving vaccination and heterologous virus challenge under different regimes of DON exposure. Pigs were divided into four groups: Group I was vaccinated with a conventional PRRSV-2 based vaccine (Ingelvac® PRRS MLV, Boehringer-Ingelheim) and infected 2 weeks later with a PRRSV-1 strain, mimicking a common condition in the field. The animals in groups II and III were analogously vaccinated and challenged, but in addition received DON during 4 weeks prior to infection, employing a daily oral administration scheme at doses of 40 and 80 µg/kg body weight, respectively (corresponding to approx. 1 and 2 mg/kg feed, respectively). This represents conditions of prolonged exposure to DON at levels that frequently occur in the field. Group IV served as control and was neither vaccinated nor treated with DON, but infected with the PRRSV-1 strain (Fig. 1). We assessed a plethora of features including DON levels, PRRSV-specific antibodies, viral presence in serum and viral load in different tissues, clinical symptoms as well as histopathological characteristics in order to determine effects of DON. We report here that exposure to DON reduced vaccine efficacy and exacerbated clinical disease upon challenge.

## Results

### DON supplementation and the effects thereof on vaccination

**Fig. 2:**
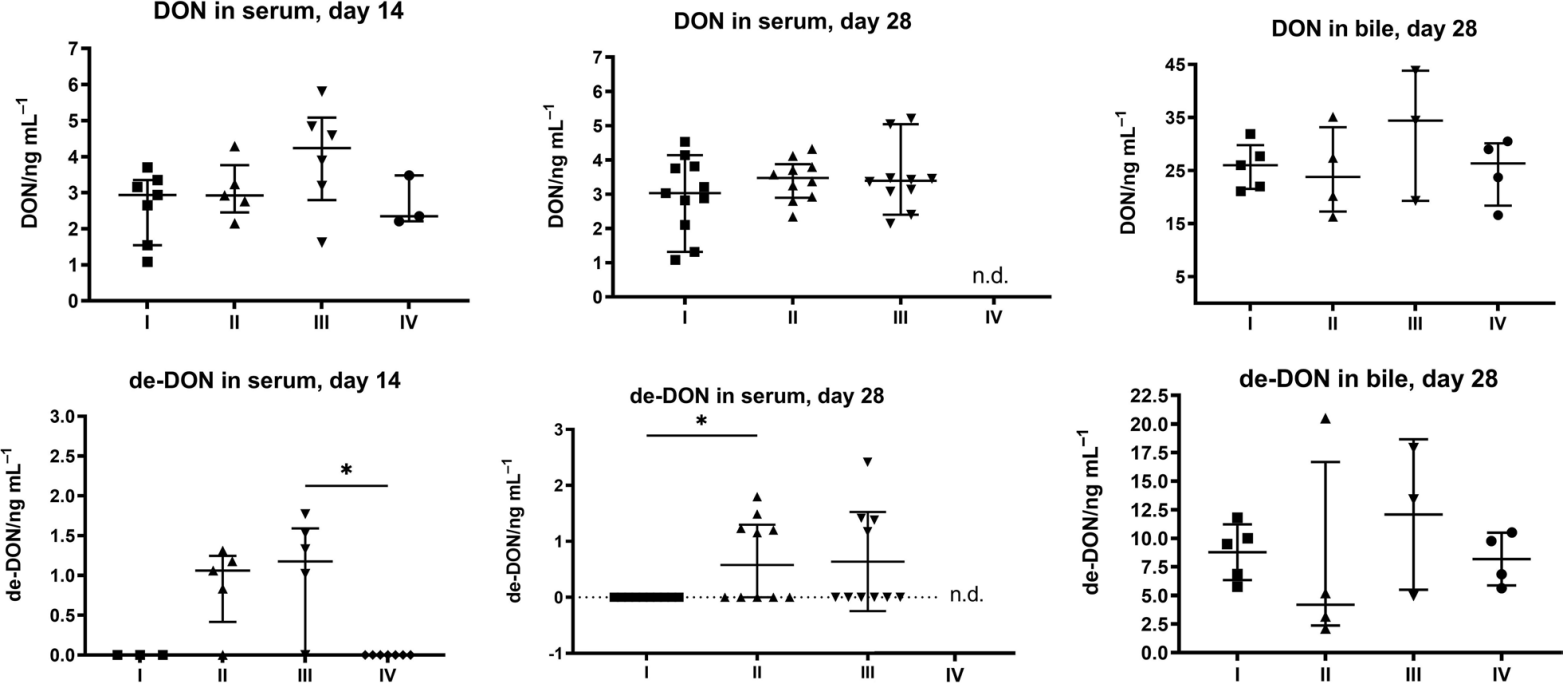
DON levels in serum and bile fluid. **A**–**C** Levels of DON (top panels) and its metabolite de-epoxy-DON (bottom panels) were determined in serum on day 14 (**A**) and day 28 (**B**) as well as in bile fluid on day 28 (**C**); n.d., not determined. Some animals were not included in the analysis for technical reasons. Each symbol represents one animal; arithmetic means are displayed as horizontal lines. Values below the limit of quantification (in the de-DON determination) were set as 0. The data were analyzed using the Kruskal–Wallis test. The graphic is shown as scatter dot blot, line at Median with interquartile range

Independently of their standard feed, the piglets in groups II and III received DON at daily doses of 40 µg/kg body weight (low-dose group II, corresponding to approx. 1 mg/kg feed) or 80 µg/kg body weight (high-dose group III, ~ 2 mg/kg feed). This was generally well-tolerated by the animals without evident signs of intoxication; only two individuals in group III sporadically vomited during the second week of DON supplementation. To verify DON uptake, we quantified DON in serum on days 14 and 28 after start of administration (Fig. 2A/B, upper panels). At these times, non-supplemented animals (groups I and IV) displayed average DON concentrations of between 2.6 and 3.2 ng/ml. DON-supplemented animals had a tendency towards elevated DON levels in blood, particularly on day 14 (3.1 ng/ml in group II and 4.0 ng/ml in group III). Differences between groups regarding DON levels in serum were lower on day 28, which might indicate adaptation to DON. However, the elevated DON exposure of animals in groups II and III is clearly evidenced by levels of the DON metabolite de-epoxy-DON (de-DON), which remained below the level of quantification in all animals of groups I and IV but was detected in the serum of 80% (day 14), Kruskal–Wallis test, statistically significant between group III and IV (*p* = 0.0282), or 40–44% (day 28) of the DON-supplemented piglets, statistically significant between group I and II (*p* = 0.0444) (Fig. 2A/B, bottom panels). In addition, we also determined DON and de-DON levels in bile fluid on day 28 and found DON levels to be elevated in response to supplementation (Fig. 2C).

The animals received commercial standard feed, mirroring common field conditions. To assess the contribution of DON naturally present in the feed, we determined its DON content; DON levels of 329 and 467.3 ng/g dry matter were measured for the two lots of feed that were employed (Denkapig Mini Start and Ferkel Start). These values are within the range of normal “background” contamination [2].

**Fig. 3:**
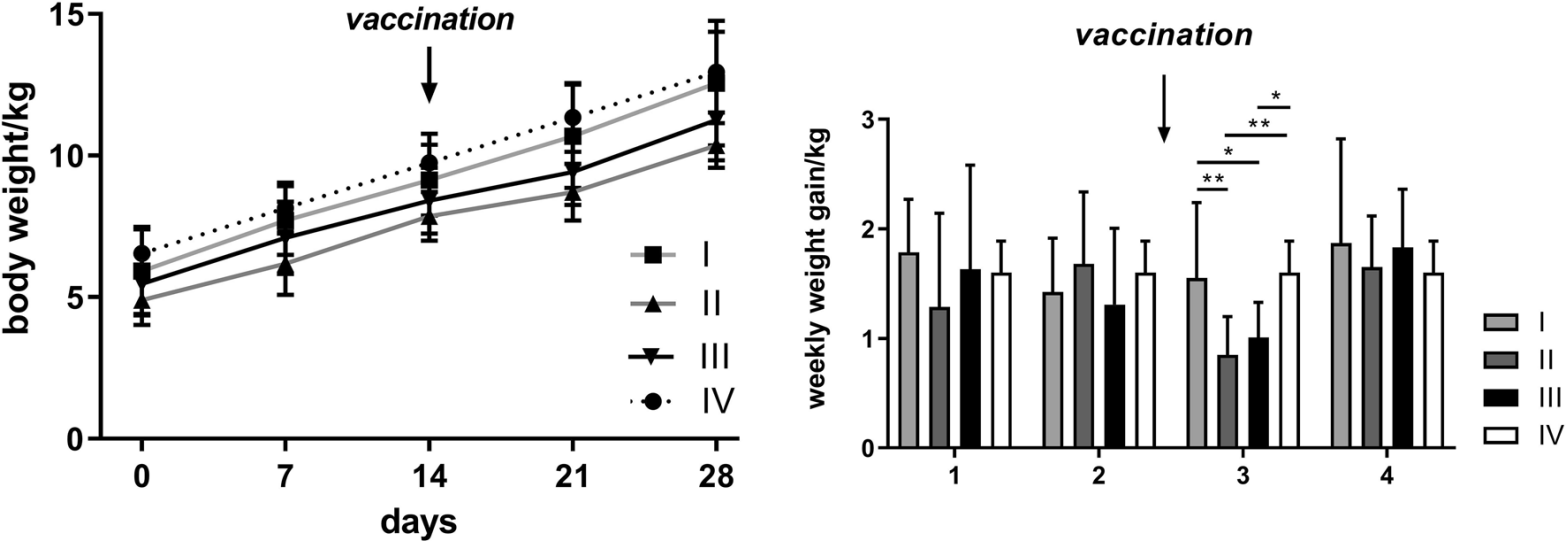
Body weight. Average body weight (mean ± SD, **A**) and average weekly weight gain (mean ± SD, **B**) of animals. The data were analyzed using one way ANOVA test. Statistically significant differences (*p* < 0.05) are described in the result section. Missing values in group IV were extrapolated linearly

The average body weight of the pigs did differ significantly between group II and IV (one-way ANOVA, *p* = 0.0380) on day 0, between group I and II (one-way ANOVA, *p* = 0.0281) and between group II and IV (one-way ANOVA, *p* = 0.0081) on day 7, between group II and IV (one-way ANOVA, *p* = 0.0072) on day 14, between group I and II (one-way ANOVA, *p* = 0.0129), II and IV (one-way ANOVA, *p* = 0.00020) and III and IV (one-way ANOVA, *p* = 0.0326) on day 21, and between group I and II (one-way ANOVA, *p* = 0.0178) and II and IV (one-way ANOVA, *p* = 0.0102) on day 28 and was thus not markedly affected by DON (Fig. 3A) despite the fact that both DON treated groups showed the lowest body weight throughout the study including day 0. In the first week after vaccination, however, the DON-supplemented animals in groups II and III gained significantly less weight (group I 1.5 kg, group II 0.85 kg, group III: 1.010 kg and group IV 1.6 kg) than the non-supplemented animals (Fig. 3B). Yet, this effect on weight gain was only transient. In total, the DON supplementation regime did not markedly reduce growth performance or affect overall health of the animals.

**Fig. 4:**
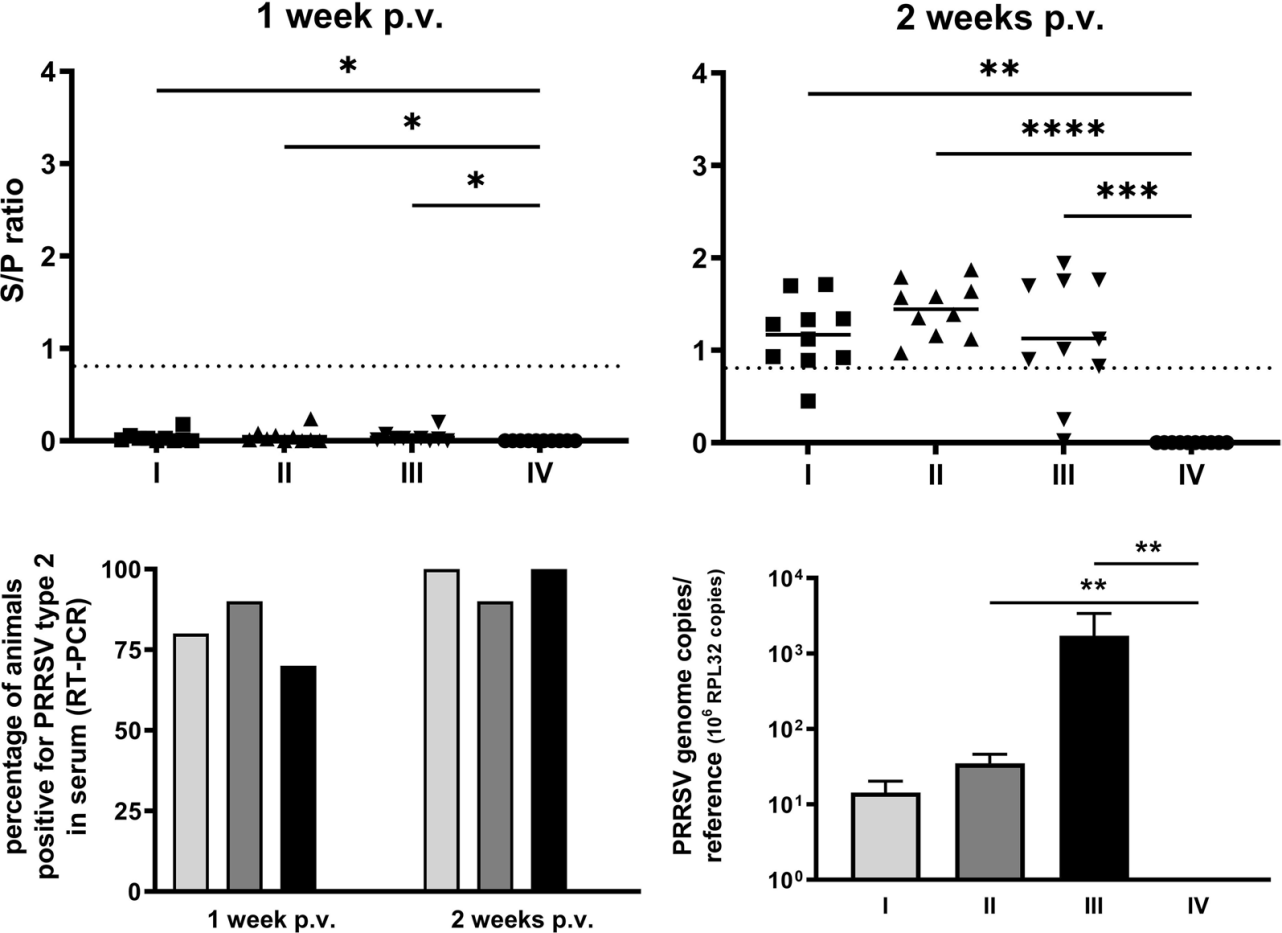
Vaccination parameters. **A**) RRSV-specific antibody titres in serum on days 21 (1 week post-vaccination, left panel) and 28 (2 weeks post-vaccination, right panel), determined by ELISA and expressed as ratio of sample/positive control (S/P). Each symbol represents one animal; median with interquartile range are displayed as horizontal lines; threshold (S/P = 0.4) is indicated with a dashed line. **B** Percentage of animals tested positive for PRRSV-2 genomes by RT-PCR. **C** Average numbers of PRRSV genome copies in lung by qRT-PCR, normalised to expression of reference gene RPL32 (mean ± SD). Statistically significant differences (Kruskal–Wallis test, *p* < 0.05) are indicated using an asterisk

Animals in groups I, II and III were vaccinated on day 14. Very mild symptoms (slightly reduced general fitness, rare events of coughing) were transiently observed in some animals without DON-related differences. We employed ELISA on serum samples to determine whether and to what extent a PRRSV-specific antibody response had been mounted (Fig. 4A). No such antibody response was seen within 7 days after vaccination (Fig. 4A, left panel, all ELISA S/P-ratios with negative result). Kruskal–Wallis test significance values are given for completeness (group I versus IV (*p* = 0.0184), group II versus IV (*p* = 0.00104), group III versus IV (*p* = 0.0126)), most vaccinated animals—but not the mock-vaccinated controls (group IV)—displayed positive ELISA signals in this analysis at 14 days post-vaccination, indicating seroconversion, Kruskal–Wallis test, group I versus IV (*p* = 0.002), group II versus IV (*p* < 0.0001), group III versus IV (*p* = 0.0010) (Fig. 4A, right panel). Remarkably however, 20% of the piglets in group III (supplemented with DON at high doses) failed to develop a PRRSV-specific antibody response within 14 days, while 100% of the vaccinated animals in groups I and II (no or low doses of DON) were clearly seropositive. While this might potentially be due to a reason unrelated to DON, the result clearly suggests an adverse effect of DON on the effectiveness of vaccination within this time frame.

We complemented this serological analysis with determinations of PRRSV by RT-PCR in serum samples (Fig. 4B). At 7 days post-vaccination, most vaccinated animals were positive for viral RNA (between 70% in group III and 90% in group II). At 14 days post-vaccination, all animals except one in group II were positive. This indicates development of viraemia in a manner that did not significantly depend on DON, Chi-square test (*p* > 0.05). In lung samples of animals sacrificed 14 days after vaccination, however, the average virus load was very low in group-I animals and clearly higher the more DON the animals had received (Fig. 4C). These differences reached a statistical significance using Kruskal–Wallis test between group IV and III (*p* = 0.0064), group IV and II (*p* = 0.0077), but not between group IV and I (*p* = 0.1389).

### Effects of DON upon virus challenge

**Fig. 5:**
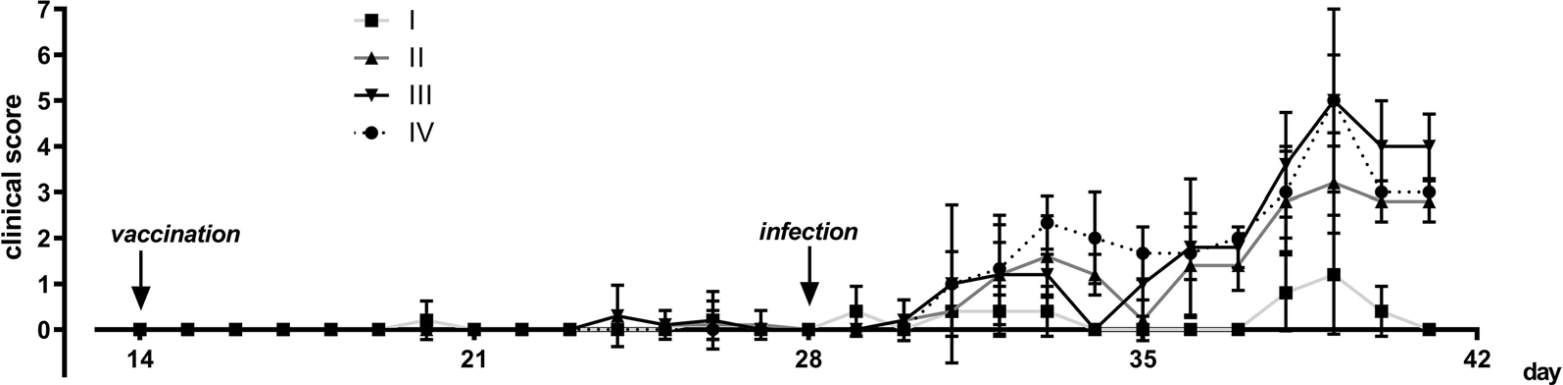
Clinical score. Disease parameters of animals in the course of the experiment. Values were 0 for all animals before vaccination (mean ± SD). The data were analyzed using the Kruskal–Wallis test. Statistically significant differences (Kruskal–Wallis test, *p* < 0.05) are described in the result section

On day 28 (14 days after vaccination), animals of all groups were challenged by infection with a PRRSV-1 field strain, genetically distant from the PRRSV-2 based vaccine. We followed the development of disease symptoms and recorded a clinical score, displayed in Fig. 5. Generally, the course of disease was relatively mild. Animals in the non-vaccinated control group (IV) developed respiratory symptoms starting on day 3 post-infection (p.i.), reaching an average disease score of 2 on day 4 p.i., followed by a transient partial recovery and aggravated disease between days 10 and 13 with disease scores of up to 5 (day 11 p.i.). In contrast, vaccinated animals (group I) did not exceed a clinical score of 1 on day 11 p.i. and were thus largely protected from disease. The piglets in the vaccinated group II (supplied with the low DON dose) displayed comparatively much more prominent symptoms with scores reaching 3 on day 11 p.i. The animals in group III, which had been vaccinated and given the high DON dose, developed disease as severely as the non-vaccinated group IV, in particular at later times, with disease scores of up to 5 (on day 11 p.i.). Thus, DON supplementation dose-dependently decreased the efficacy of the vaccine to protect against disease upon heterologous PRRSV challenge. The data were analysed using the Kruskal–Wallis test. No statistical significance was achieved on day 1 to 4 p.i. with *p *values of *p* = 0.2157, *p* > 0.99, *p* = 0.572 and *p* = 0.4489, respectively. On day 5 statistical significance was attained between group I and IV (*p* = 0.0129), but not between other groups. On day 6 significance was attained between group I and II (*p* = 0.0416), between group I and IV (*p* = 0.0192), between group II and III (*p* = 0.0414) and between group III and IV (*p* = 0.0192) but not between group I and III and between II and IV. On day 7 statistical significance was attained between group I and IV (*p* = 0.0219) and between group II and IV (*p* = 0.0793), but not between other groups. On day 8 no statistical significance between the groups was achieved. On day 9 statistical significance was attained between group I and III and (*p* = 0.0107) and between group I and IV (*p* = 0.0129), but not between other groups. On day 10 statistical significance was attained between group I and III and (*p* = 0.0183) but not between other groups. On day 11 statistical significance was attained between group I and III and (*p* = 0.0235) and between group I and IV (*p* = 0.0339), but not between other groups. On day 12 and 13 statistical significance was attained between group I and III with *p* values of *p* = 0.0014 and *p* = 0.0007, respectively, but not between other groups (Kruskal–Wallis test, *p* > 0.05).

**Fig. 6:**
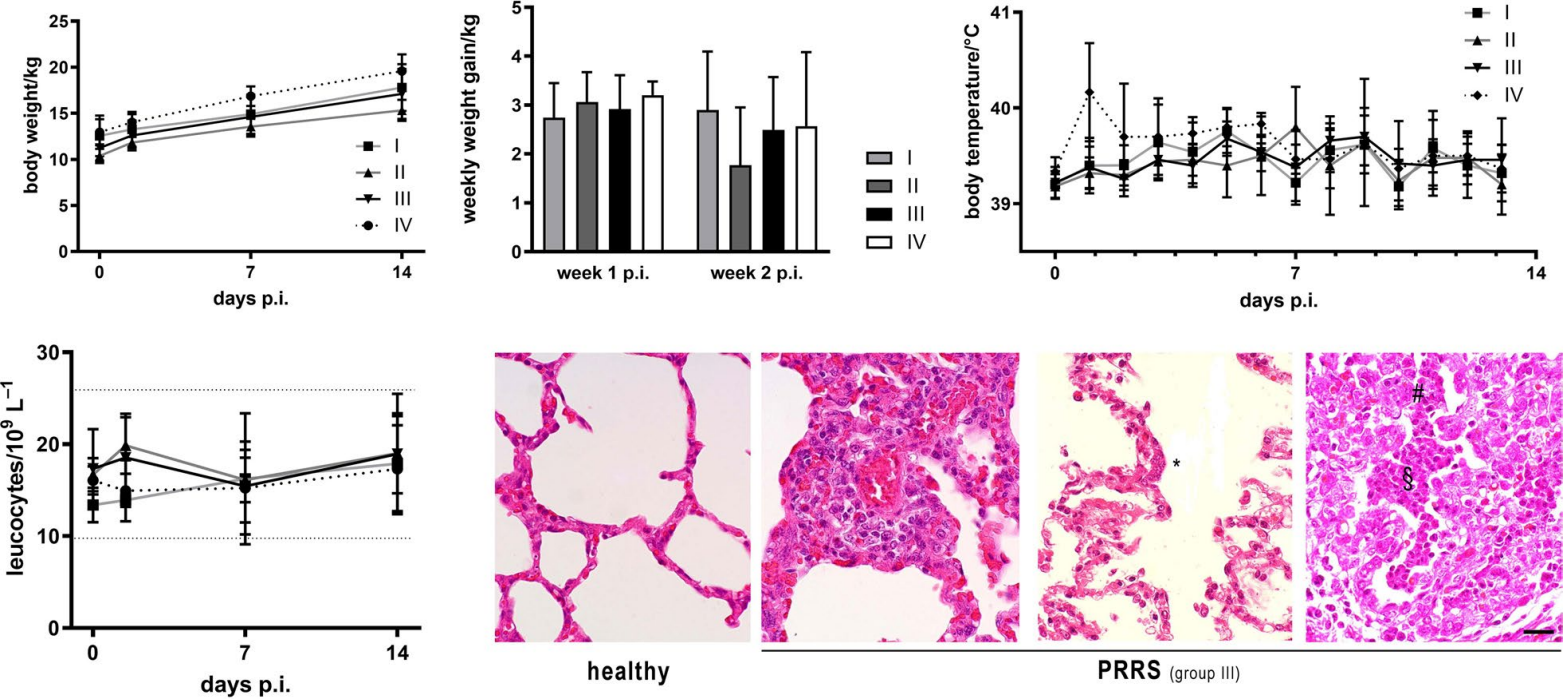
Vital parameters after infection. **A** Average body weight (left) and average weekly weight gain (right) of animals after challenge (mean ± SD). **B** Average body temperature (mean ± SD); Statistically significant differences between groups (Kruskal–Wallis test, p < 0.05) are described in the result section. **C** Total leucocyte counts in blood of animals (mean ± SD; dotted lines indicate physiological reference range). **D** Representative histological images of lung tissue of healthy animals (an animal from group IV before infection) and lung lesions seen 14 days p.i. in animals of all groups (samples from a group-III animal are shown). Typical pathological alterations include occurrence of multinucleated cells (*), alveolar exudate (§) and hypertrophy/hyperplasia of alveolar cells (#). Scale bar, 20 μm

Further, we complemented this determination of clinical scores with measurements of vital parameters as well as histopathology at the end of the experiment (14 days p.i.). Generally, the differences between the groups were not noticeable or minor. The average weekly weight gain of the animals was roughly the same in all groups; Kruskal–Wallis test in week 1 p.i. (*p* = 0.2641) and in week 2 p.i. (*p* = 0.2379) (Fig. 6A). Also, there was no large difference in average body temperatures between the groups, except for the non-vaccinated group IV showing transient fever (> 40 °C) on day 1 p.i. The data were analysed using the Kruskal–Wallis test; statistical significance was attained on day 1 and 7 between group II and IV with *p* values of *p* = 0.0471 and *p* = 0.0418, respectively. Between group III and IV and group I and IV no statistical significance was achieved. (Fig. 6B). Haematocrit values (not shown) and total leucocyte counts (Fig. 6C) were within the reference ranges throughout the experiment without any marked difference between the groups, except that there was a transient, but not significant elevation of leucocyte concentrations shortly after infection (36 h p.i.) in the DON-supplemented groups II and III (Kruskal–Wallis test, *p* > 0,05). Histopathologically, all infected animals developed characteristic changes typical for PRRS including signs of inflammation like perivascular infiltration with mononuclear cells and alveolar exudate as well as alveolar hypertrophy and hyperplasia (Fig. 6D). Such pathology was observed in all animals without significant differences between groups. Macroscopic lung lesions, in particular oedema, were observed for animals of all groups (not displayed).

**Fig. 7:**
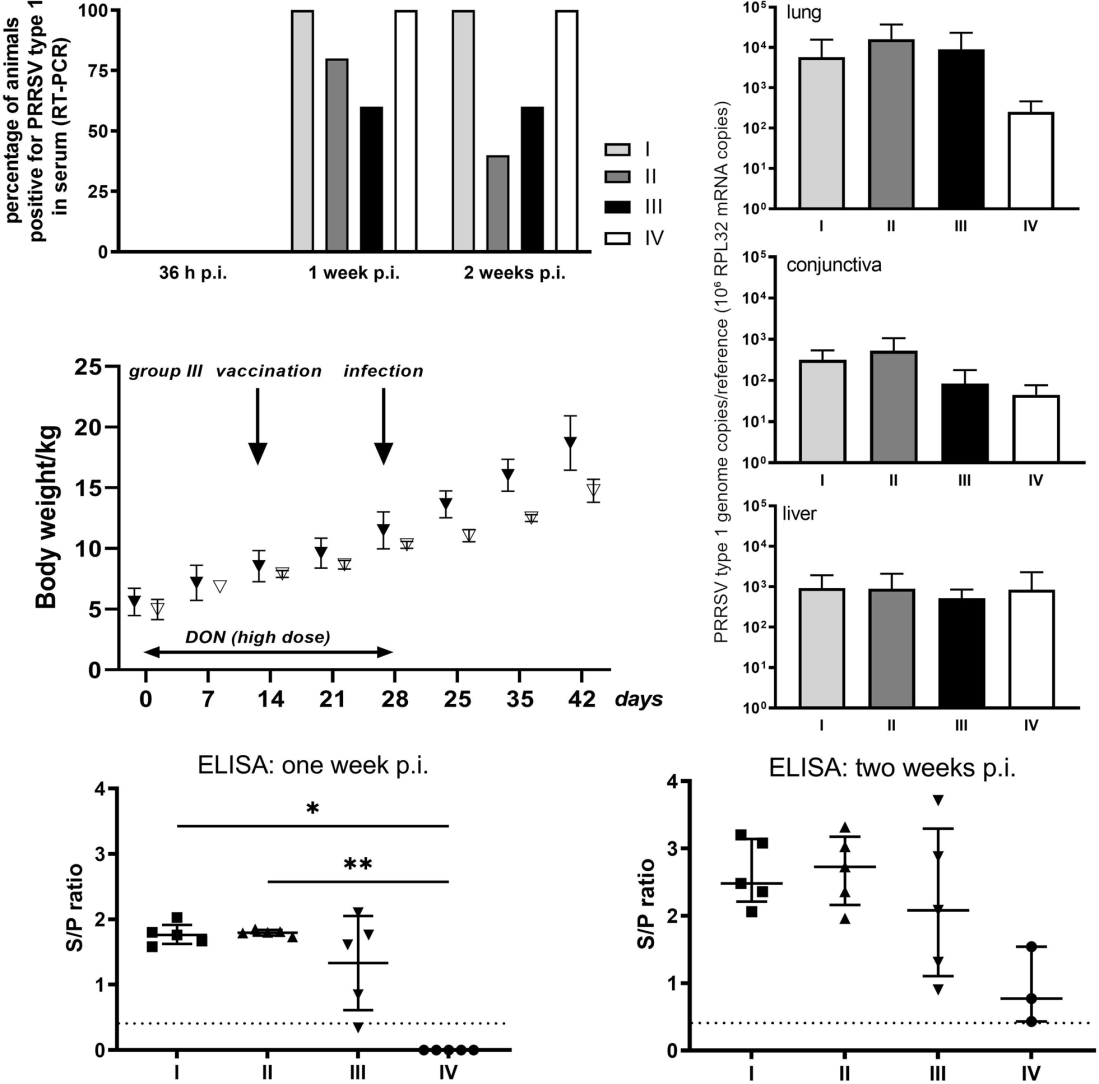
Viral parameters after infection. **A** Percentage of animals positive for PRRSV-1 (challenge strain) genomes in serum as determined by RT-PCR. Total number of animals: 5 in groups I–III, 4 in group IV. The data were analyzed using Chi-square test. **B** Average numbers of PRRSV-1 genome copies normalised to expression of reference gene RPL32 in the indicated tissues (mean ± SD). The data were analyzed using the Kruskal–Wallis test. **C** Body weight of the animals in group III (high DON dose). Filled triangles represent the animals which mount a PRRSV-specific antibody response 2 weeks post vaccination (see Fig. 4A). The values of the two piglets that failed to mount a PRRSV-specific antibody response after vaccination are displayed as open triangles. The data were analyzed using the Mann–Whitney test. **D** PRRSV-specific antibody titres in serum on days 7 (left panel) and 14 (right panel) p.i., determined by ELISA and expressed as ratio of sample/positive control (S/P). Each symbol represents one animal, means ± SD are displayed as horizontal lines; threshold (S/P = 0.4) is indicated with a dashed line. Statistically significant differences (Kruskal–Wallis test, *p* < 0.05) are indicated with an asterisk

In addition, we determined the presence of the challenge virus in serum by RT-PCR as well as the viral load in different organs using qRT-PCR. While no RNA of this virus was detectable in serum at 36 h p.i., each animal in groups I and IV (not supplemented with DON) was positive for viral RNA on day 7 p.i. and remained positive until day 14 p.i. In contrast, only a fraction of animals in the DON-supplemented groups II and III displayed viraemia at these times, Chi-square test with *p *values 1 and 2 weeks p.i. of *p* = 0.3080 and *p* = 0.1098, respectively (Fig. 7A). Further, nucleic acids of the challenge virus were found in lung tissue samples of all groups, taken at the end of the experiment (14 days p.i.), albeit with a tendency towards lower viral loads in the control group IV (Fig. 7B, top panel; normalised to expression of the cellular reference gene RPL32). This indicates presence of viral RNA in the respiratory tract of all animals in all groups. In conjunctiva (Fig. 7B, middle panel), the amount of viral nucleic acids was generally much lower than in the lung, with highest levels measured for groups I and II. In samples of liver, where PRRSV is typically less prevalent, viral loads were low and not different between groups (Fig. 7B, bottom panel). Also, there was no significant difference between the animals of group III (Mann–Whitney test, *p* > 0.05) where no PRRSV-specific antibodies had been induced within 2 weeks after vaccination and the other animals in this group. Yet, such differences were noticed when looking at the body weight of the individual group-III animals: After challenge infection (but not before), the piglets that had remained seronegative upon vaccination displayed the lowest body weights within the group (up to 18% below the average; Fig. 7C). This might point towards an intensification of the impact of PRRSV on animal health as a consequence of DON exposure.

Lastly, we measured total anti-PRRSV antibody titres by ELISA to evaluate the extent of adaptive immune response (Fig. 7D). All animals in the non-vaccinated control group IV developed PRRSV-specific antibodies within 14 days after infection. Antibody titres of the vaccinated animals were generally higher. However, the average titres for group III, which had received the high DON dose, remained lower than those of groups I and II (statistical significance was attained using Kruskal–Wallis test between group I and IV (*p* = 0.021) and between group II and IV (*p* = 0.0033). The group-III animals that had remained seronegative within 2 weeks after vaccination also developed anti-PRRSV antibodies upon challenge infection to similar levels as the other piglets in this group. This indicates that the high-dose DON supplementation (which ceased on the day of challenge infection) did not irreversibly impede the ability to mount a PRRSV-specific immune response in these animals.

## Discussion

 In this animal trial, we evaluated the influence of the common mycotoxin DON on vaccination with a typical PRRSV-2 MLV, followed by challenge with a PRRSV-1 field strain. We found that upon administration of the high DON dose of 80 µg/kg body weight (~ 2 mg DON/kg feed, group III), 20% of animals failed to mount a detectable antibody response to PRRSV within 2 weeks after immunisation. This may be highly relevant in field conditions, where such concentrations of DON are frequently encountered in the pigs’ feed [2]: Success of PRRSV vaccination appears to be impaired by DON contamination. Of note, DON levels in this study did not detectably affect overall fitness or the average daily weight gain of the animals—thus, the adverse effect of DON on vaccine efficacy may even occur at “unsuspiciously low” contamination levels, below the threshold where adverse effects on growth rates would become obvious to the farmer. In the light of our results, it appears very unlikely that overall stress or general lack of fitness was the reason for the partial failure of the vaccine at high DON doses (group III). As late as 14 days post-vaccination, viral loads in the lung were highest in this group, which points towards a potential delay of the porcine immune system to clear the attenuated vaccine virus from the lung and/or a delay in virus replication at higher DON levels.

Upon heterologous challenge with a PRRSV-1 field strain of intermediate pathogenicity, vaccinated animals were basically protected from disease, but less so if they had been exposed to DON. Disease severity in the vaccinated animals that were supplemented with the high DON dose (group III) was as high as in the non-vaccinated control group (IV), again showing that the protective effect of PRRSV vaccination was severely compromised by DON. While there were no major differences between the groups with respect to vital parameters such as body weight gain or viral load in various tissues, the total PRRSV-specific antibody titres remained lower in group III (vaccinated + high DON dose) than the other vaccinated groups. This again suggests some adverse effects of DON on the porcine immune system, at least with respect to PRRSV. Such interference of DON might concern the cellular immune response in particular; Already upon supplementation with low doses of DON (group II), clinical disease was clearly aggravated while antibody titres remained unaffected in comparison to group I. However, we did not perform in-depth immunological analysis of e.g. T cell responses, which remains to be done in a follow-up study.

During completion of this study, Savard et al. published that DON decreased PRRSV replication in cell culture already at non-cytotoxic levels [24], that DON-contaminated feed exacerbated disease induced by PRRSV in pigs [25] and that DON dose-dependently impaired induction of PRRSV-specific antibodies upon vaccination with a PRRSV MLV [26]. Hallmarks of viral replication were reduced upon DON administration in all these studies. In combination, these data basically corroborate the results reported here, further supporting our conclusions. We are, however, the first to report adverse effects of DON in vivoin a PRRSV vaccination–challenge system.

Some of the adverse effects of DON observed here were less pronounced than those in the in vivo studies conducted by Savard et al. [25, 26]. In those studies however, higher doses of DON were used (up to 3.5 mg/kg feed, whereas the highest dose in our study was selected to approximate 2 mg/kg feed). Also, only the most prevalent *Fusarium* toxin DON, but no other mycotoxin was varied in our study, while Savard et al. employed naturally DON-contaminated feed rather than pure DON administration separately. Natural infection of grain with *Fusarium* molds also generates several other mycotoxins in addition to DON [2]; these are likely to exacerbate adverse effects of DON in a synergistic manner. Most notably, the experimental diets used by Savard et al. [25, 26] contained zearalenone (ZEN) concentrations higher than the critical level of 0.1 mg/kg diet for female piglets [5]. ZEN is known as a potent endocrine disruptor and also interferes with the immune system [27].

*Ad libitum* feeding of DON-contaminated diets (as performed by Savard et al., [25, 26]) typically leads to low blood levels that persist for long periods of time, whilst the bolus application of pure DON (as in our study) is expected to result in a sharp peak (t_max_) at between < 1 and 4 h after administration, followed by distribution and elimination. Blood base-line levels should be reached approximately 30 h after t_max_, considering a terminal half-life of approximately 6 h [28]. Therefore, the blood levels in the DON-exposed animals in this study, measured 24 h after the last DON bolus, represent the stable phase of elimination. Even under these conditions, the targeted differences in DON exposure could be demonstrated for the DON-supplemented groups II and III, where DON and de-DON levels were generally higher than in the non-exposed groups. The low DON levels that were detected in the control groups (I, IV) are likely due to the background contamination of the pigs’ feed with DON, which is inevitable. The animals were kept without straw bedding, excluding another potential source of intoxication with mycotoxins.

Despite differences in experimental design, both we and others [25, 26] observed inhibitory effects of DON with respect to PRRSV biology in vivo. However, these properties cannot be readily generalised to all pathogens. In fact, another relevant porcine pathogen, porcine circovirus-2 (PCV2), was found to replicate *better* at low doses of DON in cell culture, albeit without significant exacerbation of disease in vivo [29]. Thus, at least some effects of DON appear to be specific to PRRSV. Future studies will have to show by what mechanism(s) DON and other mycotoxins specifically influence features of PRRSV replication as well as the immune response to this virus. Of note, PRRSV infection is specially known to delay and subdue the adaptive immune response of the host [22, 23]. This may favour immune impairment by DON—even at subtoxic levels, which are frequently found in feed and were mimicked in this study. Even though some effects reported in this study did not reach statistical significance (mostly due to high variations between individual animals within a group, as frequently observed in studies involving pigs), the overall tendency of the results strongly suggests adverse effects of DON on PRRSV vaccine efficacy and disease. Future studies will have to show by what mechanism(s) DON and other mycotoxins specifically influence features of PRRSV replication as well as the immune response to this virus.

Since it is practically impossible to completely prevent *Fusarium* infection of crops, efforts to decontaminate feed [2, 30] may be highly relevant to ensure efficacy of PRRSV vaccination.

## Conclusions

The experimental vaccination/challenge study revealed that exposure of pigs to DON at subtoxic levels (approx. 1 or 2 mg DON/kg feed) for a period of 4 weeks deteriorates the efficacy of vaccination against clinical signs of PRRS. Therefore DON might represent a risk factor to be considered regarding PRRSV biology in vivo and PRRSV vaccine efficacy under natural field conditions.

## Materials and methods

### Animals

Thirty-eight healthy crossbred piglets (German Landrace × Pietrain) of either sex were obtained at the age of 3 weeks from the Oberholz farm for teaching and research, University of Leipzig, Germany. Piglets were vaccinated against porcine circovirus-2 (PCV2, CircoFLEX ®, Boehringer-Ingelheim) on day 20 after birth. All animals were free of PRRSV and other major porcine pathogens (including influenza virus, porcine circovirus, porcine parvovirus, data not shown). The piglets were housed separately in a fully air-conditioned biosafety level 2 stable on a slatted floor at the University of Leipzig, Faculty of Veterinary Medicine. They were randomly distributed into four groups as outlined in Fig. 1 and described below. Animals had free access to water and feed (Denkapig Mini Start and Ferkel Start, LHG Schmölln, Germany) throughout the experiment.

Animals were monitored daily and weighed weekly. Blood samples were taken on days 0, 7, 14, 21, 28, 29.5, 35 and 42 from the vena cava cranialis employing the Vacuette system (Greiner Bio-One, approx. 7 ml blood/sample) and prepared for serology and PCR analyses (see below). Rectal body temperature was measured daily starting on day 28 (prior to infection). Half of the animals (i.e., 5 animals in groups I–III and 4 animals in group IV) were euthanised and sectioned on day 28 to obtain pre-challenge organ and bile samples; the remaining animals were euthanised on day 42 (14 days post-infection). Samples were stored at − 20 °C (sera) or − 80 °C (organs) until analysis (see below). One piglet in group IV was withdrawn and excluded from the study on day 35 due to its severely compromised health condition, characterised in particular by elbow joint inflammation and thus unrelated to PRRS.

For euthanasia, animals were anaesthesised with 2 mg/kg body weight azaperon (Stresnil, Janssen-Cilag) and 20 mg/kg ketamine (Ursotamin, Serumwerk Bernburg, Germany) injected i.m., followed by intraveneous (i.v.) administration of 0.1 ml/kg T61 (Intervet).

 This study was fully approved by the local animal welfare authority (Regierungspräsidium Leipzig), file number TVV02/13.

### DON administration

Crystalline deoxynivalenol (DON, generously provided by University of Hohenheim, Institute of Animal Nutrition, Stuttgart, Germany) was solubilised in sterile water and administered orally as a daily bolus to each individual animal in group II and III on days 1–28 (prior to infection). Doses were adjusted to 40 µg/kg body weight (“low dose”, corresponding to approx. 1 mg/kg feed; group II) and 80 µg/kg body weight (“high dose”, corresponding to approx. 2 mg/kg feed; group III), respectively.

### Vaccination

All animals in groups I, II and III (10 animals/group) were vaccinated on day 14 after weaning by intramuscular (i.m.) administration of a 2-ml dose of the commercial PRRSV-2 based Ingelvac® PRRS MLV (Boehringer-Ingelheim) into the neck muscle. Animals in group IV (control group, 8 animals) were mock-vaccinated with 0.9% NaCl on day 14.

### Challenge virus

All animals were infected intranasally on day 28 (i.e., 2 weeks post-vaccination) with the German PRRSV-1 field isolate Cobbelsdorf (Virus collection of the Federal Research Institute for Animal Health, Insel Riems, Germany, no. 210, [31]). One-millilitre doses of 1 × 10^5^ TCID_50_ were administered with a syringe into each nostril. The virus had been tested in a pilot assay to be infectious in vivo and to induce PRRS-specific clinical signs under these conditions (data not shown).

The virus was grown and titrated on MARC-145 cells (ATCC CRL-12,231), cultured as described [32]. For virus propagation, cells were inoculated with virus for 60 min in Dulbecco’s modified Eagle’s medium (DMEM) without additives, followed by incubation in DMEM with 1% foetal bovine serum (FBS) for 5–7 days until development of cytopathic effect. Subsequently, flasks were frozen (− 80 °C) and thawed, followed by harvesting of the supernatant by centrifugation (210×*g*, 5 min). Virus was aliquotted and stored at − 80 °C. Virus titrations were performed by immunofluorescence using an in-house serum from a PRRSV-positive pig. Virus titres were calculated as TCID_50_/ml according to Spearman and Kärber [33].

### Clinical score

Animals were monitored daily for development of disease, focussing on respiratory symptoms. A clinical score was determined for each animal, taking into account the following criteria, each of which was assessed with a value between 0 (healthy) and 3 (severe disease): general condition (vivid—listless); respiration rate (0 = regular at < 18 min^− 1^; 1 = regular at > 18 min^− 1^; 2 = regular at 26–35 min^− 1^ or irregular at < 35 min^− 1^; 3 = frantic at > 35 min^− 1^); coughing (0 = none; 1 = less than 2 times in 30 min; 2 = 3–5 times in 30 min; 3 = more than 5 times in 30 min); sneezing (none—more than 5 times in 30 min); nasal discharge (none—severe); ocular discharge; conjunctival oedema; cyanosis.

### Determination of viral load and antibody titres

Viral RNA was isolated from sera and cell culture supernatants with ZR Viral RNA kit (Zymo Research). RNA isolation from tissue samples was done with RNeasy kit (Qiagen) using a TissueLyser/QIAcube system (Qiagen) according to the manufacturer’s instructions. RNA was reverse-transcribed with SuperScript III (Thermo), employing oligo(dT)_18_ primers. Quantitative (real-time) PCR after reverse transcription (qRT-PCR) was performed with the SYBRgreen-based RotorGene system (Qiagen) using primer pairs 5**′**-AAAGAAAAGTACAGCTCCGATGGGGA + TCCTCCCCTAGGTTGCTGGCG (designed and validated to be specific for open reading frame (ORF) 7 of the PRRSV-1 strain Cobbelsdorf, GenBank JN651692.1), CATCGCTCAGCAAAACCAGTCCAG + GACAGACACAATTGCCGCTCACTAG (ORF7 of the PRRSV-2 strain VR-2332, the origin strain of Ingelvac® PRRS MLV, GenBank AY256686.1) or TGCTCTCAGACCCCTTGTGAAG + TTTCCGCCAGTTCCGCTTA (cellular reference gene ribosomal protein L32 (RPL32) [10]). Results were normalised to the reference gene RPL32 wherever applicable (i.e., in organ samples).

PRRSV-specific antibody titres in sera were determined using the commercial HerdChek PRRS X3 ELISA kit (IDEXX) according to the manufacturer’s instructions. The ratio of sample/positive control (S/P) was employed for assessment of titres; S/P > 0.4 was considered positive.

### Mycotoxin determination

DON and its metabolite de-epoxy-DON (de-DON) were quantified in serum and bile as described [34, 35]. In brief, both serum and bile was incubated over night with the enzyme β-glucuronidase (Type H-2 from *Helix pomatia*, Sigma-Aldrich). Afterwards, the serum samples were purified by solid phase extraction (Oasis HLB, Waters), while bile samples were subjected to clean-up by immunoaffinity chromatography (DZT MS-Prep, R-Biopharm). Subsequently, the analytes were determined by liquid chromatography-coupled mass spectrometry (LC-MS/MS) [34]. The limits of quantification for DON and de-DON were 0.45 and 0.76 ng/ml in serum and 0.15 and 0.08 ng/ml in bile with mean recoveries of 105 and 131% in serum and 95 and 96% in bile, respectively. Results were not corrected for recoveries. DON levels of feed were analysed by high-performance liquid chromatography (HPLC) with diode array detection after sample purification with immuno-affinity columns (DONprep, R-biopharm) as described [36].

### Data analysis and statistics

Data display was performed with GraphPad Prism (version 49.20). Data were also employed for statistical analysis using one way ANOVA test, Kruskal–Wallis text, Chi-square test or Mann–Whitney test as indicated. For all tests, *p* < 0.05 was considered statistically significant.

## Data Availability

All data generated or analysed during this study are included in this published article [and its supplementary information files].

## Abbreviations

DON: Deoxynivalenol
ELISA: Enzyme-linked immunosorbent assay
i.m.: Intramuscular
MAPK: Mitogen-activated protein kinase
MLV: Modified live vaccine
p.i.: Post-infection
p.v.: Post-vaccination
PRRS(V): Porcine reproductive and respiratory syndrome (virus)
(q)-PCR: (Quantitative) polymerase chain reaction
RT: Reverse transcription
TCID_50_: 50% Tissue culture infectious dose
